# Chronic Intestinal Stasis from the Viewpoint of the Internist**Oration delivered at the 41st annual meeting of the Alumni Association of the medical department of the Univsersity of Buffalo, May 30-June 2, 1916.

**Published:** 1916-08

**Authors:** Charles D. Aaron

**Affiliations:** Professor of Gastro-Enterology in the Detroit College of Medicine and Surgery, Detroit, Michigan


### BUFFALO MEDICAL JOURNAL

Yearly Volume 72 AUGUST, 1916 Number 1

### ORIGINAL ARTICLES

**The right is reserved to decline papers not dealing with practical medical and surgical subjects, and such as might offend or fail to interest readers. Contributors are solely responsible for opinions, methods of expression and revision of proof.**

### Chronic Intestinal Stasis From the Viewpoint of the Internist.**

By CHARLES D. AARON, Sc. D., M. D.,

Professor of Gastro-Enterology in the Detroit College of Medicine and Surgery, Detroit, Michigan.

Lane* employs the term "chronic intestinal stasis" to indicate such abnormal delay in the passage of the intestinal contents through a portion or portions of the gastro-intestinal tract as to result in the absorption into the circulation of a greater quantity of toxic or poisonous materials than can be disposed of by the liver or other protective organs. He believes this condition is due to the upright position of the trunk, which induces a prolapse of the viscera (gastroenteroptosis), and consequent faulty drainage. To resist this displacement, nature reduplicates certain peritoneal tissues, which become firmer and firmer until distinct bands are formed, holding up the terminal ileum and causing a kink, with stasis and toxemia. It is assumed that the intestinal toxemia or the mechanical irritation caused by the tugging of the displaced organs on the mesenteries will result in the formation of pericolic membranes, bands, adhesions, folds, kinks and angulations of sufficient number to cripple the peristaltic function. The kinks occur at the duodeno-jejunal juncture, terminal ileum, cecal region, hepatic flexure, splenic flexure, and colon-sigmoid juncture. By surgical intervention he has succeeded in curing coincident pyorrhea alveolaris,

*W. Arbuthnot Lane. The Lancet. Dec. 21, 1912, page 1706.
**Oration delivered at the 41st annual meeting of the Alumni Association of the medical department of the University of Buffalo, May 30-June 2, 1916.

tuberculosis, arthritis deformans, nephritis, systitis, pyelitis, endometritis, salpingitis, exophthalmic goitre, skin diseases, colitis, endocarditis, epilepsy, neurasthenia, and a host of other diseases.

I dislike to appear wanting in appreciation of the great and valuable service which modern surgery is rendering to the human race, but I do wish to submit some convictions which have become strong in me, in view of the recent developments that have caught the fancy of practitioners and evoked expectations in the laity which I cannot help but deem as hazardous on the part of the former as it may turn out to be delusive on the part of the latter. I will not say what I have heard from many, that there is much unnecessary surgery nowadays, for that would be quite false and inappreciative of the obvious good which is done by conscientious and efficient men. Nor will I urge, as again some do, that operations are advised and performed under the stress of professional preoccupation and enthusiasm, for I have too high an opinion and from my own personal observation too clear a judgment of the profound scrupulousness of surgeons to want even a word of mine to go toward lending aid to the insinuation; but I do believe that not infrequently surgeons yield much upon a general theory, deferring to and following a name and a precedent, and thus exhibiting both the weakness of human nature and the fallacy of incomplete science.

I propose to speak of gastroenteroptosis under the new name chronic intestinal stasis, and of the futility of the operative treatment of it. I have nothing but respect, the highest respect, for the achievements of modern surgery. As its technic becomes more certain, its service for good will become incalculable. But because it is so fascinating and so wonderful in effect, the surgeon is frequently misled into urging it, and the patient into blindly trusting it. It is the internist to whom the patient, weak from his operation, comes in search of final relief. It is the internist who appreciates the fact that many an apparently successful operation entails serious disabilities which diminish the patient's efficiency and in many cases impair his health to such an extent that he never can resume his place as a man of service to the community. And very often it is the internist's conviction that conservative methods, slow but sure, would have served more reliably than operative intervention.

A Roentgen ray examination with the bismuth mixture may show a displaced stomach, a prolapsed colon, kinking of the hepatic or splenic flexures, spasms of different loops of the intestine, the presence of bands, membranes and adhesions; but such conditions do not imply that surgery is inevitably necessary. So long as motility is not interfered with, there is no absolute indication for surgical intervention. A transverse colon can be displaced anywhere from its normal position down to the symphysis without interfering with motility. The cinematograph shows that such a displaced intestine can empty itself properly even if the angulations at the distal ileum and the hepatic and splenic flexures show absolute kinks. It has been proved that stasis is not due to an abnormal position of the intestine (kink, ptosis, or redundant colon) so long as there is no actual mechanical obstruction. What busy surgeon follows up these cases and knows just what becomes of them, other than that they left him after the successful operation? What does he know of the disabilities the patient has had to accept as the price of his recovery, disabilities which reduce his earning capacity and force him to change the whole trend of his life?

One of the operations to which patients are now frequently subjected unnecessarily is gastroenterostomy. To be sure, a gastroenterostomy, when indicated, brings about astonishing results, especially in the case of an obstruction of the pylorus. But no operation has been more abused and thus become the cause of more harm. Its use can never be justified as a cure for gastroenteroptosis and intestinal stasis. Besides this operation we find surgeons anchoring a displaced kidney, attaching the colon to the abdominal wall, suspending the stomach in a hammock made of the great omentum, plicating the gastrohepatic and gastrophrenic ligaments, suturing the gastrocolic omentum to the anterior abdominal wall, and resorting to many other so-called original expedients for the cure of kinks causing intestinal stasis. Others are sure that ileocolostomy, the short-circuiting of the ileac contents directly into the sigmoid, is the best treatment. It has been found that after this operation the entire colon may show a tendency to fill. To obviate this difficulty the operation of extirpation of the colon or colectomy is practised. This is not a simple operation; even in the hands of skilled surgeons the mortality is high.

The futility of these operations is clear when we recall that in over 90 per cent. of cases of gastroenteroptosis the position of the abdominal viscera is a normal conformation to the physical status of the body. Patients suffering from such a characteristic form do not respond to surgical intervention. It is a general condition of relaxation, which is not helped by extirpation or short-circuiting of any part of the colon. These patients develop into neurasthenics and suffer from nervous dyspepsia associated with gastric and intestinal atony. The anatomic dislocation of the organs is of minor importance compared with the pathologic condition of the digestive nervous system. It is to be hoped that with a better understanding of the pathologic anatomy of ptosis the **furor operativus** will be broken and yield to internal medicine.

Recent experimental work by Keith* explains the mechanism of intestinal movements, and seems to account for the production of intestinal stasis upon a physiologic basis. In his histological studies of the mechanism of the heart beat, he investigated different portions of the intestine of various animals in the hope of finding the mechanism controlling involuntary muscle. As a result of extensive research along these lines he discovered a nodal tissue intermediate between nerve and muscle and interposed between Auerbach's myenteric plexus and the smooth muscle of the intestinal wall. This intermediate tissue, consisting of branched cells in direct connection with both nervous and muscular elements, bears the same relation to the intestinal musculature as the primitive nodes and conducting tissues of the heart bear to the auricular and ventricular muscular masses. It possesses two distinct functions: one, the initiation and regulation of the muscular contractions in the segment of the intestine which it controls; the other, the power of conducting impulses which lead to the forward propulsion of the intestinal contents. Notable collections of this ''nodal and conducting'' tissue were found in the region of the cardia of the stomach, where it initiates gastric movements; near the ampulla of Vater, to control the movements of the pylorus and duodenum; a lesser collection near the beginning of the jejunum, exercising the same function over the greater portion of the remaining small intestine; in the region of the ileo-colic valve, to control the lower ileum and proximal portion of the colon; and others in the transverse, descending, and iliac colon, and in the rectum.

Not only do the anatomic sites and the demonstrable physiologic functions of these ''nodes'' explain the normal movements of the intestine, but it is obvious that a perversion of the function of any one of them is capable of giving rise to an inhibition of the forward progress of the intestinal contents, with resulting intestinal stasis. In the establishment of this as the physiologic explanation of the mechanism of the production of intestinal stasis, Keith* was able to demonstrate the presence of definite fibrotic and degenerative changes in this nodal tissue in segments of the intestine removed by Lane and others for the relief of chronic intestinal stasis.

Keith found that food passing along the alimentary tract is

*New York Medical Journal, Sept. 11, 1915, page 573.

*Arthur Keith. A New Theory of the Causation of Enterostasis. The Lancet, August 21, 1915, page 375.

propelled through a series of zones or segments furnished with their own pacemakers. There are two such zones in the heart—an auricular and a ventricular; the sino-auricular node is the master pacemaker. A block of imperfection in conduction may occur between these two zones of the heart, with the result that "back-pressure"—a venous stasis—is produced. Similar irregularities may occur in the nodal and conducting system of the alimentary canal. When they do occur we find them at the joints where one rhythmical zone or area passes into another. A block may occur where the esophagus joins the stomach; another where the gastric zone ends and the duodenal begins; where the duodenal zone passes into the jejunal, and where the iliac passes into the colic. A block may occur at any point of passage from a lower to a higher rhythm.

It is clear that to obtain an orderly propulsion of the food along the whole length of the alimentary canal these various rhythmic zones must be closely coordinated in their action; and this coordination means a complicated system of reflexes. Disturbance in any one segment upsets the rhythm in all the segments. We know it is a law of the intestine that distention of one part inhibits the action of the part below it. It is easy to see that disturbance in the excitability of the pacemaker of the cecum will be reflected to the lower ileum. One can therefore easily understand on the hypothesis of Keith how stasis in the colon may be followed by ileal, or stasis in the duodenum by gastric stasis, and how the disturbance of the conductivity or excitability of the rhythmic zone may ultimately give rise to stasis in the whole intestine. From this we are compelled to believe that the musculature of the alimentary tract is the sole propelling power in the intestinal wall, and that a defect in the intestinal musculature must play a part in the causation of chronic intestinal stasis. From his investigations Keith concludes that it is improbable that mechanical conditions or derangements of sphincteric action underlie the production of intestinal stasis, but that the true cause is the production of some "block" or disorder in the nodal and conducting system of the intestine analogous to the heart block and other similar disturbances of cardiac function. He does not accept Lane's "drag, band and kink" theory.

Many cases of chronic intestinal stasis prove after operation to have been nothing but spastic constipation. The retardation in the evacuation of the bowel is induced by a spasm of one or more isolated loops of the intestine. These spasms occur most frequently at the hepatic and splenic flexures, sigmoid, rectum and anus. Enterospasm is brought about by an increased irritability of the autonomic nervous system which may be due to neuropathic conditions associated with diseases of the abdominal viscera or pelvic organs or to vagotonia associated with neurasthenia and hysteria. The Roentgen ray plainly shows the contracted loop of intestine, and its constant presence leads one to believe it to be organic. When making a roentgenographic diagnosis, the use of atropine hypodermically is of great assistance in preventing the error of sending the patient to the operating table.

Some surgeons have reported the constant presence of demonstrable mechanical obstruction in cases of chronic intestinal stasis, while others of equal ability have frequently reported obstruction without any distinct mechanical factor whatever. An operation like that of colectomy is an extensive and dangerous one, and seems hardly justifiable in the treatment of such chronic joint diseases as arthritis deformans and of tuberculosis. It is surprising and a bit confusing to hear pretensions of cure of so many varied and unrelated diseases, such as the followers of Lane make with regard to the same remedial operation. There is a lack of sense of proportion in the connection which is made between intestinal stasis and the many forms of ill-health, and in the magnified importance which is attached to the all-saving power of the short-circuiting operation. In view of the radical treatment urged by the followers of Lane, and the extreme confidence placed in its not yet entirely tested results, internists will do well to cultivate a sane and safe conservatism. It seems unwarranted to encourage and stimulate surgeons to hazard the performance of major operations without a most circumspect antecedent employment of all possible internists' aids and cautions. In considering the end results of ileocolostomy in the treatment of chronic intestinal stasis, I can do no better than quote the findings of James T. Case* who has had special advantages for the roentgenologic study of patients after gastric and intestinal operations. He says, "I have been especially fortunate in having been able to study a large series of cases after ileocolostomy performed for adhesions, ileal stasis, Lane's kink of the ileum, and other nonmalignant lesions. Forty such cases have been examined very carefully, operation in most of them having been performed according to the technic advocated by Lane, one of them by Lane himself. Not all the patients in this series were clinically failures, though the majority of them were examined for the cause of unsatisfactory result. In most of them, during the early months following operation, the result was apparently worth while, and in a few striking in-

*Journal of the American Medical Association, Nov. 6, 1915, page 1632.

stances the operation had seemed to work like magic; but sooner or later, usually within a year after the surgical procedure, the symptoms have begun to recur and the patient's last state is worse than the first. It should be remembered that this series of cases represents the work of more than a dozen different surgeons, most of them of national reputation —work which I believe to be on the whole representative of the best in technic which this country affords. The unsatisfactory results can, therefore, hardly be attributed to poor technic."

The treatment of chronic intestinal stasis is not a matter of plumbing. Drainage plays an important part, but not "human plumbing," as some surgeons have called it. The intestine is not a thick rigid tube, depending upon gravity for its emptying. This has been proved by Cannon and Blake in their experimental work on gastroenterostomy. The food and liquids invariably pass through the pylorus after the operation of gastroenterostomy unless the pylorus be occluded.

The short-circuiting operation or the removal of the colon is proper only when there is a very decided obstruction to the passage of the intestinal contents and in such cases the clinical symptoms of obstruction are easily recognizable. Most patients suffering from chronic intestinal stasis will respond to medical treatment. Conservative methods are coming into favor because of their evident and many-sided merits. They comprise dietetic, physical and medicinal treatment, combined with the accurate fitting of an abdominal bandage.

The internal treatment of chronic intestinal stasis is practically the same as that for gastroenteroptosis. It should be directed toward the improvement of general nutrition in order to counteract the neurasthenia and to strengthen the muscles of the abdominal walls. Patients who are poorly nourished must be well fed. The diet should be as nutritious as possible; it should contain a large proportion of fats and oils which form laxative soaps. Sometimes it is necessary to resort to "forced feeding."

Hydrotherapeutic measures may be instituted in conjunction with the diet. Systematic respiratory gymnastics are to be carried out several times a day. The muscles of the body may be stimulated by dry rubbing of the skin with rough towels. Cold water, half-baths, and the Scotch douche on the abdomen, with rubbing and slapping, are valuable. Massage plays an important role in strengthening the abdominal muscles. If a bag of salt weighing from one to five pounds be placed on the abdomen, the efforts of the patient to elevate the abdomen with this weight will strengthen the muscles.

The mechanical treatment consists principally in bandaging the abdomen with a suitable support to the relaxed abdominal wall. It acts beneficially by ameliorating the symptoms which arise from tension or stretching of the mesenteries. As to the bandage I may say that I devised a model in 1897* which I have employed with almost uniform success in a variety of cases. The wearing of such a bandage not only raises the position of the abdominal organs, but relieves the strain and tugging on the nerves in the mesenteries. In this way the normal function of the organs is re-established. In other words, we restore normal physiology and obtain a bodily condition which readily responds to dietetic, physical and medicinal treatment.

All patients suffering from chronic intestinal stasis require iron in some form. They cannot take it internally, owing to its irritating effect on the gastric mucous membrane. This difficulty can be overcome by the intramuscular medication.

Many cases of chronic intestinal stasis recover completely when the accompanying constipation is properly treated. It is of the utmost importance to decide whether the constipation is of the atonic or the spastic type. The differentiation is not always easy. Patients suffering from spastic constipation are vagotonic. This can be easily recognized by the positive oculo-cardiac reflex, by Herring's phenomenon, and by the pilocarpine test. Spastic constipation is due to constriction or spasm of a few isolated loops of the intestine, readily demonstrable by the Roentgen ray. The fluoroscope will also show the relaxing effect of a hypodermic of atropine upon the spasm. The enterospasm may be painful or not; in the former case it is due to neuropathic conditions associated with diseases of the abdominal viscera or pelvic organs. Atonic constipation is due to abdominal tonus followed by relaxation and atony of the intestinal musculature.

The aim of the treatment of the atonic variety of constipation must be to so improve the muscular condition by dietetic measures as to finally attain regularity of defecation with a normal supply of food. The diet should be large and bulky, rich in insoluble residue, usually an increased amount of carbohydrate, and more particularly of foods rich in cellulose.

The small quantity of fecal matter present in the intestines of individuals suffering from chronic intestinal stasis is due to loss of water from prolonged retention of the feces, and also to an extraordinarily effective utilization of the food.

---

*Charles D. Aaron, Gastroptosis, Journal of the American Medical Association, July 31, 1897.

This utilization is too effective; the bacteria of putrefaction and fermentation cannot flourish in so poor a medium as the exhausted feces. Consequently the intestine is deprived of the stimulus of large quantities of fermenting and putrefactive products, with the result that motility is retarded. The therapeutic indication is to render the feces more voluminous, softer and more liquid, so as to bring into play the physiologic stimulation of peristalsis. Schmidt found that agar would fulfill the above-mentioned indications.

Agar is hard to digest, but absorbs and retains moisture, thus permanently increasing its own volume. It resists the action of intestinal bacteria as well as that of the enzymes, and gives bulk to the feces without introducing objectionable products of decomposition. Agar is not a purgative, but belongs to the diet in intestinal stasis just the same as fruits and vegetables rich in cellulose. Since it is harmless, its use may be continued for months.

In the treatment of the spastic variety of constipation, bulky foods are eliminated and a variety of fruits should be given because of their chemical constitution. They stimulate peristalsis, partly because of their fruit acids, and partly because they contain sugar, which tends to increase the fermentative processes in the intestine. Easily melted fats, as well as butter, oil and cream, not only have a mechanical effect, but also act chemically, stimulating peristalsis by means of the great amount of fatty acids they develop.

Petroleum jelly will lubricate the whole gastro-intestinal tract, thus facilitating the passage of the contents. The lubrication of the chyme in the intestine assists in its timely removal in cases of intestinal stasis. After the due administration of this jelly the feces are softened and under the microscope are found to contain minute oil globules. Petroleum jelly of the best quality seems to act better than the Russian mineral oil; it is heavier and therefore mixes more thoroughly with the feces; at the same time its viscosity prevents it from passing through the bowel too rapidly. The jelly, when pure, is not absorbed from the alimentary tract and even in large doses has no poisonous effect. It is useful not only as a lubricant, but also as a means of healing superficial lesions of the mucous membrane.

The sovereign remedy in spastic constipation is atropine or belladonna. The extract of belladonna may be given in doses of .008Gm. (1/8 grain) three or more times daily until the physiologic action of the drug is secured. The medication is to be continued until the throat becomes dry, when the dose should be gradually diminished. Phenolphthalein in a dose a 0.2 Gm. (3 grains) can be safely combined with the belladonna. This combination can be used as a gauge of the amount of belladonna to be taken. If the patient is instructed to diminish the number of doses when the bowels move too freely, there will be little or no danger of belladonna poisoning.

There is no treatment that has given me better results in chronic intestinal stasis than Fleiner's oil cure. This consists in injecting into the rectum once daily 250 to 500 mils (½ to 1 pint) of olive oil. I have used pure cottonseed oil with equal benefit. The oil is to be retained in the bowel for a considerable time; it is best to retain it over night if possible. These injections are continued several months, at first daily, later every other day, and subsequently twice a week. In cases of spastic constipation the oil has a soothing effect on the mucous membrane, thus relieving spasm; and in atonic constipation it strengthens the muscular tonus. Besides, it lubricates the gut, softens fecal agglomerations, and forms a protective layer upon the damaged mucous membrane.

When there is putrefaction, lactic acid may be prescribed, best in the potential form of the Bulgarian bacillus. The cultures are available in liquid and tablet form. The efficacy of this treatment is enhanced by a diet preponderantly carbohydrate and by restriction of proteins. The intestinal flora should show a change after a few days.

Transduodenal lavage, one to four pints of water being introduced through the duodenal tube, as suggested by Jutte, will often give good results. To inhibit the growth of the anaerobic bacteria, oxygen can be introduced through the duodenal tube.

38 West Adams Avenue.

---

**Cause of Bilharziosis.** F. G. Cawson, Pietermaritzburg, Med. Jour. of S. Africa, Feb. 1916. The ovum of Schistosomum Japonicum is oval or globular and spineless. Ova with a lateral spine, are found in Brazil, Puerto Rico, Egypt and tropical Africa, in the bowel affection. In Egypt, the lateral-spined egg in the faeces is associated with the terminal-spined egg in the urine. In S. Africa, only terminal-spined eggs are found, both in the bowel affection and in haematuria. The adult parasite cannot be differentiated between the varieties producing the terminal and lateral-spined ova. The Japanese have traced the infection from cercariae in the liver of snails, in stagnant pools, to the mesenteric systems of mice; also from merocidia in infected cattle to cercariae in the livers of snails. Various bathing pools in S. Africa rivers have long been popularly believed to be sources of infection and the author and others have found infected snails in some of these waters.

